# (3*E*,5*E*)-3,5-Bis(4-hy­droxy­benzyl­idene)oxan-4-one

**DOI:** 10.1107/S1600536810051457

**Published:** 2010-12-11

**Authors:** Zhi-Yun Du, Hua-Rong Huang, Yu-Jun Lu, Kun Zhang, Yan-Xiong Fang

**Affiliations:** a Guangdong University of Technology, Faculty of Chemical Engineering and Light Industry, Guangzhou 510006, Guangdong, People’s Republic of China

## Abstract

In the title compound, C_19_H_16_O_4_, there are two 4-hy­droxy­benzyl substituents on the oxan-4-one (tetra­hydro­pyran-4-one) ring, which exhibits an envelope conformation. The dihedral angles between pyran­one ring and the two benzene rings are 26.69 (9) and 36.01 (9)° while the benzene rings make a dihedral angle of 20.88 (10)°. In the crystal, mol­ecules are linked by inter­molecular O—H⋯O hydrogen bonds into a supra­molecular three-dimensional twofold inter­penetrating hydrogen-bonded network.

## Related literature

For the pharmacological activity or curcumin [systematic name (1*E*,6*E*)-1,7-bis(4-hydroxy-3-methoxyphenyl)-1,6-hep­ta­diene-3,5-dione], see: Maheshwari *et al.* (2006 ▶). For curcumin analogues, see: Liang *et al.* (2009 ▶). For the synthesis of the title compound, see: Youssef *et al.* (2004 ▶); Du *et al.* (2006 ▶). For related structures, see: Abaee *et al.* (2008 ▶); Du *et al.* (2006 ▶).
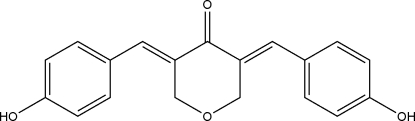


## Experimental

### 

#### Crystal data

C_19_H_16_O_4_
*M* *_r_* = 308.32
Orthorhombic, 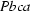

*a* = 11.812 (3) Å
*b* = 7.4687 (16) Å
*c* = 33.233 (7) Å
*V* = 2931.9 (11) Å^3^
*Z* = 8
Mo *K*α radiation
μ = 0.10 mm^−1^
*T* = 293 K
0.42 × 0.37 × 0.29 mm

#### Data collection

Bruker SMART CCD 1K area-detector diffractometer
Absorption correction: multi-scan (*SADABS*; Sheldrick, 1996 ▶) *T*_min_ = 0.960, *T*_max_ = 0.972
16659 measured reflections
3224 independent reflections
1941 reflections with *I* > 2σ(*I*)
*R*_int_ = 0.053

#### Refinement

*R*[*F* ^2^ > 2σ(*F* ^2^)] = 0.045
*wR*(*F* ^2^) = 0.124
*S* = 1.04
3224 reflections
211 parameters
H-atom parameters constrained
Δρ_max_ = 0.17 e Å^−3^
Δρ_min_ = −0.18 e Å^−3^

### 

Data collection: *SMART* (Bruker, 2000 ▶); cell refinement: *SAINT-Plus* (Bruker, 2000 ▶); data reduction: *SAINT-Plus*; program(s) used to solve structure: *SHELXS97* (Sheldrick, 2008 ▶); program(s) used to refine structure: *SHELXL97* (Sheldrick, 2008 ▶); molecular graphics: *SHELXTL* (Sheldrick, 2008 ▶); software used to prepare material for publication: *SHELXTL*.

## Supplementary Material

Crystal structure: contains datablocks global, I. DOI: 10.1107/S1600536810051457/jh2219sup1.cif

Structure factors: contains datablocks I. DOI: 10.1107/S1600536810051457/jh2219Isup2.hkl

Additional supplementary materials:  crystallographic information; 3D view; checkCIF report

## Figures and Tables

**Table 1:** Hydrogen-bond geometry (Å, °)

*D*—H⋯*A*	*D*—H	H⋯*A*	*D*⋯*A*	*D*—H⋯*A*
O3—H3⋯O4^i^	0.82	1.95	2.757 (2)	167
O4—H4⋯O1^ii^	0.82	1.86	2.677 (2)	171

Symmetry codes: (i) 
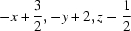
; (ii) 
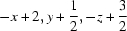
.

**Fig. 1.**
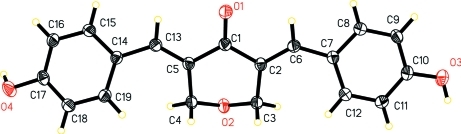
Perspective view showing 30% probability displacement ellipsoids and the atom-numbering scheme.

**Fig. 2.**
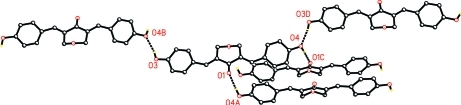
Hydrogen bonds of the title compound via the O—H···O are shown as dashed lines. Symmetry: A = -x + 2, y - 1/2, -z + 3/2; B = -x + 3/2, -y + 2, z -1/2; C = -x + 2, y + 1/2, -z + 3/2; D = -x + 3/2, -y + 2, z + 1/2.

**Fig. 3.**
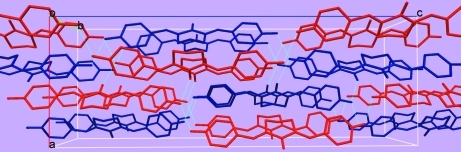
Crystal packing of the title compound, viewed along the b axis, showing the three dimensional two-fold interpenetrating hydrogen bonding network. Dashed lines indicate hydrogen bonds.

## supplementary crystallographic information

### Comment

For thousands of years Eastern medicine has used curcumin, the major component of turmeric, for a wide range of health benefits, but only in recent times has its biological action been understood. Curcumin possesses a wide spectrum of pharmacological activities including anti-oxidant, anti-inflammatory, antiviral, antifungal, cancer chemo preventive, cancer chemotherapeutic properties (Maheshwari *et al.*, 2006). As the limitation of solubility, stability and activity of curcumin for clinical application, many series of curcumin analoges with a monoketone function have attracted interests in trial of improving the properties (Liang *et al.*, 2009). This class of compounds is readily synthesized by reacting a substituted benzaldehyde with tetrahydropyran-4-one; in the case of the title compound 4-hydroxybenzaldehyde was used as the reactant.

The compound was purified by re-crystallization from THF and characterized by NMR spectrum and ESI mass spectrum. The analytical and spectroscopic data are consistent with the proposed structure given in Scheme 1. The molecular structure of the title compound contains the two 4-methylbenzyl substituents on the tetrahydropyran-4-one and the six-member ring adopts an envelope conformation with the flap oxygen atom displaced by 0.648 (8) Å from the plane of the other five atoms (Figure 1).

Similar structures have been observed in the literature (Abaee *et al.*, 2008; Du *et al.*,2006).

The dihedral angles formed between the mean plane through the six atoms of the pyranone ring and two benzene rings of 4-methylbenzyl groups are 26.69 (9) and 36.01 (9)°, the corresponding dihedral angles between two benzene rings of 4-methylbenzyl groups is 20.88 (10) °.

In the crystal packing, intermolecular O—H···O hydrogen bonds (Figure 2, table 1) connect the molecules into a supramolecular three-dimensional two-fold interpenetrating hydrogen bonding network (Figure 3).

### Experimental

The title compound was synthesized using a general procedure (Du *et al.*,2006; Youssef *et al.*, 2004) 4-hydroxybenzaldehyde (0.01 mol) and tetrahydropyran-4-one (0.005 mol) were dissolved in THF and added 0.5 mL concentrated HCl as catalyst. The mixture was warmed at 298-303 K for 12 h, cold water was added to precipitate the yellow compound. Crystals were obtained by recrystallization from THF. The formulation was established by the NMR spectrum and ESI mass spectrum. ^1^H NMR (MSDO-d^6^, 300 MHz) δ (ppm): 10.03 (brs, 2H, -COH), 7.55 (s, 2H, -CCH=), 7.28 (d, J = 8.1, 4H, ArH), 6.85 (d, J = 8.1, ArH), 4.85 (s, 4H, -CCH_2_-O-CCH_2_-C). The ESI mass spectrum showed ions at 308.

### Refinement

The C-bound H atoms were positioned geometrically and were included in the refinement in the riding-model approximation, with distances 0.96 (CH_3_), 0.97 (CH_2_) and 0.95 Å (aromatic); *U*_iso_(H) = 1.2Ueq(C) for H atoms on secondary and tertiary C atoms, and *U*_iso_ = 1.5Ueq(C) for methyl H atoms. The two water H atoms were located in a difference Fourier map and then refined as riding on the water O atom with *U*_iso_(H) = 1.5Ueq(O).

### Crystal data

**Table:**

C_19_H_16_O_4_	*F*(000) = 1296
*M**_r_* = 308.32	*D*_x_ = 1.397 Mg m^−^^3^
Orthorhombic, *P**b**c**a*	Mo *K*α radiation, λ = 0.71073 Å
Hall symbol: -P 2ac 2ab	Cell parameters from 3683 reflections
*a* = 11.812 (3) Å	θ = 2.5–26.7°
*b* = 7.4687 (16) Å	µ = 0.10 mm^−^^1^
*c* = 33.233 (7) Å	*T* = 293 K
*V* = 2931.9 (11) Å^3^	Block, colourless
*Z* = 8	0.42 × 0.37 × 0.29 mm

### Data collection

**Table:**

Bruker SMART CCD 1K area-detector diffractometer	3224 independent reflections
Radiation source: fine-focus sealed tube	1941 reflections with *I* > 2σ(*I*)
graphite	*R*_int_ = 0.053
φ and ω scans	θ_max_ = 27.1°, θ_min_ = 1.2°
Absorption correction: multi-scan (*SADABS*; Sheldrick, 1996)	*h* = −15→14
*T*_min_ = 0.960, *T*_max_ = 0.972	*k* = −8→9
16659 measured reflections	*l* = −42→40

### Refinement

**Table:**

Refinement on *F*^2^	Secondary atom site location: difference Fourier map
Least-squares matrix: full	Hydrogen site location: inferred from neighbouring sites
*R*[*F*^2^ > 2σ(*F*^2^)] = 0.045	H-atom parameters constrained
*wR*(*F*^2^) = 0.124	*w* = 1/[σ^2^(*F*_o_^2^) + (0.0545*P*)^2^ + 0.5774*P*] where *P* = (*F*_o_^2^ + 2*F*_c_^2^)/3
*S* = 1.04	(Δ/σ)_max_ = 0.001
3224 reflections	Δρ_max_ = 0.17 e Å^−^^3^
211 parameters	Δρ_min_ = −0.18 e Å^−^^3^
0 restraints	Extinction correction: *SHELXL97* (Sheldrick, 2008), Fc^*^=kFc[1+0.001xFc^2^λ^3^/sin(2θ)]^-1/4^
Primary atom site location: structure-invariant direct methods	Extinction coefficient: 0.0029 (5)

### Special details

**Table:**

Geometry. All esds (except the esd in the dihedral angle between two l.s. planes) are estimated using the full covariance matrix. The cell esds are taken into account individually in the estimation of esds in distances, angles and torsion angles; correlations between esds in cell parameters are only used when they are defined by crystal symmetry. An approximate (isotropic) treatment of cell esds is used for estimating esds involving l.s. planes.
Refinement. Refinement of F^2^ against ALL reflections. The weighted R-factor wR and goodness of fit S are based on F^2^, conventional R-factors R are based on F, with F set to zero for negative F^2^. The threshold expression of F^2^ > 2sigma(F^2^) is used only for calculating R-factors(gt) etc. and is not relevant to the choice of reflections for refinement. R-factors based on F^2^ are statistically about twice as large as those based on F, and R- factors based on ALL data will be even larger.

### Fractional atomic coordinates and isotropic or equivalent isotropic displacement parameters (Å^2^)

**Table:**

	*x*	*y*	*z*	*U*_iso_*/*U*_eq_	
C1	0.91919 (16)	0.7445 (3)	0.61879 (5)	0.0348 (4)	
C2	0.88020 (14)	0.8252 (2)	0.58058 (5)	0.0322 (4)	
C3	0.79877 (16)	0.9793 (3)	0.58346 (5)	0.0374 (5)	
H3A	0.8083	1.0564	0.5602	0.045*	
H3B	0.7219	0.9337	0.5831	0.045*	
C4	0.79701 (16)	0.9771 (3)	0.65410 (5)	0.0374 (5)	
H4A	0.7206	0.9298	0.6536	0.045*	
H4B	0.8042	1.0530	0.6776	0.045*	
C5	0.87964 (15)	0.8251 (2)	0.65713 (5)	0.0323 (4)	
C6	0.92068 (15)	0.7571 (3)	0.54608 (5)	0.0342 (4)	
H6	0.9721	0.6642	0.5496	0.041*	
C7	0.89904 (14)	0.8015 (2)	0.50413 (5)	0.0327 (4)	
C8	0.97612 (15)	0.7402 (3)	0.47550 (6)	0.0386 (5)	
H8	1.0364	0.6693	0.4839	0.046*	
C9	0.96621 (15)	0.7811 (3)	0.43528 (6)	0.0404 (5)	
H9	1.0188	0.7372	0.4169	0.048*	
C10	0.87774 (16)	0.8876 (3)	0.42219 (6)	0.0395 (5)	
C11	0.79695 (16)	0.9439 (3)	0.44952 (6)	0.0440 (5)	
H11	0.7354	1.0111	0.4408	0.053*	
C12	0.80732 (16)	0.9009 (3)	0.48974 (6)	0.0410 (5)	
H12	0.7519	0.9389	0.5077	0.049*	
C13	0.91900 (14)	0.7598 (3)	0.69197 (5)	0.0340 (4)	
H13	0.9732	0.6703	0.6894	0.041*	
C14	0.89027 (15)	0.8069 (2)	0.73343 (5)	0.0322 (4)	
C15	0.96953 (15)	0.7720 (3)	0.76362 (5)	0.0366 (5)	
H15	1.0367	0.7144	0.7569	0.044*	
C16	0.95080 (15)	0.8209 (3)	0.80322 (6)	0.0387 (5)	
H16	1.0048	0.7966	0.8228	0.046*	
C17	0.85055 (15)	0.9063 (3)	0.81330 (6)	0.0381 (5)	
C18	0.76801 (16)	0.9319 (3)	0.78463 (6)	0.0401 (5)	
H18	0.6989	0.9821	0.7919	0.048*	
C19	0.78746 (15)	0.8832 (2)	0.74526 (6)	0.0373 (5)	
H19	0.7311	0.9014	0.7262	0.045*	
O1	0.98058 (13)	0.6110 (2)	0.61862 (4)	0.0528 (4)	
O2	0.81578 (11)	1.08088 (17)	0.61899 (4)	0.0395 (3)	
O3	0.87617 (13)	0.9311 (2)	0.38255 (4)	0.0589 (4)	
H3	0.8139	0.9718	0.3766	0.088*	
O4	0.83014 (11)	0.9651 (2)	0.85156 (4)	0.0542 (4)	
H4	0.8896	0.9992	0.8618	0.081*	

### Atomic displacement parameters (Å^2^)

**Table:**

	*U*^11^	*U*^22^	*U*^33^	*U*^12^	*U*^13^	*U*^23^
C1	0.0388 (10)	0.0336 (10)	0.0321 (10)	0.0021 (8)	−0.0001 (8)	−0.0024 (9)
C2	0.0376 (10)	0.0295 (10)	0.0294 (10)	−0.0016 (8)	0.0009 (8)	0.0011 (8)
C3	0.0434 (11)	0.0372 (11)	0.0316 (11)	0.0040 (8)	0.0004 (9)	0.0003 (9)
C4	0.0475 (11)	0.0368 (11)	0.0279 (10)	0.0060 (8)	0.0027 (8)	0.0010 (8)
C5	0.0360 (10)	0.0315 (10)	0.0295 (10)	−0.0003 (8)	−0.0007 (8)	−0.0016 (8)
C6	0.0369 (10)	0.0320 (10)	0.0338 (11)	0.0030 (8)	−0.0009 (8)	0.0013 (9)
C7	0.0375 (10)	0.0308 (10)	0.0299 (10)	−0.0007 (8)	0.0011 (8)	−0.0006 (8)
C8	0.0386 (11)	0.0413 (11)	0.0361 (11)	0.0057 (9)	−0.0003 (8)	−0.0013 (9)
C9	0.0412 (11)	0.0471 (12)	0.0328 (11)	0.0019 (9)	0.0066 (9)	−0.0055 (9)
C10	0.0441 (11)	0.0427 (12)	0.0317 (11)	−0.0042 (9)	−0.0012 (9)	0.0017 (9)
C11	0.0453 (12)	0.0467 (12)	0.0401 (12)	0.0122 (9)	−0.0050 (9)	0.0005 (10)
C12	0.0394 (11)	0.0494 (12)	0.0342 (11)	0.0065 (9)	0.0028 (8)	−0.0030 (9)
C13	0.0372 (10)	0.0321 (10)	0.0329 (11)	0.0014 (8)	−0.0011 (8)	−0.0041 (9)
C14	0.0378 (10)	0.0294 (10)	0.0295 (10)	−0.0004 (8)	−0.0018 (8)	−0.0016 (8)
C15	0.0356 (10)	0.0385 (11)	0.0359 (11)	0.0026 (8)	0.0006 (8)	−0.0028 (9)
C16	0.0382 (11)	0.0472 (12)	0.0307 (11)	−0.0009 (9)	−0.0059 (8)	−0.0019 (9)
C17	0.0436 (11)	0.0402 (11)	0.0306 (11)	−0.0048 (9)	0.0028 (9)	−0.0077 (9)
C18	0.0372 (11)	0.0451 (12)	0.0379 (11)	0.0022 (9)	0.0036 (9)	−0.0029 (10)
C19	0.0370 (11)	0.0412 (11)	0.0336 (11)	0.0002 (8)	−0.0038 (8)	0.0017 (9)
O1	0.0708 (10)	0.0518 (9)	0.0357 (8)	0.0293 (8)	−0.0031 (7)	−0.0019 (7)
O2	0.0563 (9)	0.0305 (7)	0.0316 (7)	0.0050 (6)	0.0022 (6)	0.0008 (6)
O3	0.0632 (10)	0.0830 (12)	0.0305 (8)	0.0091 (9)	−0.0008 (7)	0.0092 (8)
O4	0.0501 (9)	0.0778 (11)	0.0346 (8)	−0.0016 (8)	0.0036 (7)	−0.0213 (8)

### Geometric parameters (Å, °)

**Table:**

C1—O1	1.233 (2)	C10—O3	1.357 (2)
C1—C2	1.479 (2)	C10—C11	1.383 (3)
C1—C5	1.485 (2)	C11—C12	1.380 (3)
C2—C6	1.343 (2)	C11—H11	0.9300
C2—C3	1.503 (2)	C12—H12	0.9300
C3—O2	1.418 (2)	C13—C14	1.462 (2)
C3—H3A	0.9700	C13—H13	0.9300
C3—H3B	0.9700	C14—C15	1.397 (2)
C4—O2	1.418 (2)	C14—C19	1.398 (2)
C4—C5	1.501 (3)	C15—C16	1.383 (2)
C4—H4A	0.9700	C15—H15	0.9300
C4—H4B	0.9700	C16—C17	1.386 (3)
C5—C13	1.340 (2)	C16—H16	0.9300
C6—C7	1.455 (2)	C17—O4	1.367 (2)
C6—H6	0.9300	C17—C18	1.376 (3)
C7—C8	1.394 (2)	C18—C19	1.377 (2)
C7—C12	1.398 (2)	C18—H18	0.9300
C8—C9	1.376 (3)	C19—H19	0.9300
C8—H8	0.9300	O3—H3	0.8200
C9—C10	1.383 (3)	O4—H4	0.8200
C9—H9	0.9300		
			
O1—C1—C2	120.58 (16)	O3—C10—C11	123.72 (18)
O1—C1—C5	121.12 (17)	O3—C10—C9	116.96 (17)
C2—C1—C5	118.27 (16)	C11—C10—C9	119.32 (18)
C6—C2—C1	117.91 (17)	C12—C11—C10	120.24 (18)
C6—C2—C3	124.92 (16)	C12—C11—H11	119.9
C1—C2—C3	117.18 (15)	C10—C11—H11	119.9
O2—C3—C2	111.83 (14)	C11—C12—C7	121.60 (18)
O2—C3—H3A	109.2	C11—C12—H12	119.2
C2—C3—H3A	109.2	C7—C12—H12	119.2
O2—C3—H3B	109.2	C5—C13—C14	130.28 (18)
C2—C3—H3B	109.2	C5—C13—H13	114.9
H3A—C3—H3B	107.9	C14—C13—H13	114.9
O2—C4—C5	111.52 (14)	C15—C14—C19	117.14 (16)
O2—C4—H4A	109.3	C15—C14—C13	118.46 (16)
C5—C4—H4A	109.3	C19—C14—C13	124.39 (16)
O2—C4—H4B	109.3	C16—C15—C14	121.80 (17)
C5—C4—H4B	109.3	C16—C15—H15	119.1
H4A—C4—H4B	108.0	C14—C15—H15	119.1
C13—C5—C1	119.01 (17)	C15—C16—C17	119.20 (17)
C13—C5—C4	123.99 (16)	C15—C16—H16	120.4
C1—C5—C4	116.99 (15)	C17—C16—H16	120.4
C2—C6—C7	132.00 (17)	O4—C17—C18	118.33 (17)
C2—C6—H6	114.0	O4—C17—C16	121.56 (17)
C7—C6—H6	114.0	C18—C17—C16	120.10 (18)
C8—C7—C12	116.55 (17)	C17—C18—C19	120.19 (18)
C8—C7—C6	117.65 (16)	C17—C18—H18	119.9
C12—C7—C6	125.80 (16)	C19—C18—H18	119.9
C9—C8—C7	122.31 (18)	C18—C19—C14	121.31 (17)
C9—C8—H8	118.8	C18—C19—H19	119.3
C7—C8—H8	118.8	C14—C19—H19	119.3
C8—C9—C10	119.82 (17)	C3—O2—C4	111.76 (14)
C8—C9—H9	120.1	C10—O3—H3	109.5
C10—C9—H9	120.1	C17—O4—H4	109.5
			
O1—C1—C2—C6	−5.0 (3)	O3—C10—C11—C12	−176.96 (19)
C5—C1—C2—C6	176.83 (17)	C9—C10—C11—C12	2.8 (3)
O1—C1—C2—C3	175.61 (18)	C10—C11—C12—C7	0.6 (3)
C5—C1—C2—C3	−2.6 (2)	C8—C7—C12—C11	−3.3 (3)
C6—C2—C3—O2	−148.76 (18)	C6—C7—C12—C11	176.68 (19)
C1—C2—C3—O2	30.6 (2)	C1—C5—C13—C14	−176.43 (17)
O1—C1—C5—C13	5.4 (3)	C4—C5—C13—C14	3.7 (3)
C2—C1—C5—C13	−176.41 (16)	C5—C13—C14—C15	−156.10 (19)
O1—C1—C5—C4	−174.69 (18)	C5—C13—C14—C19	24.7 (3)
C2—C1—C5—C4	3.5 (2)	C19—C14—C15—C16	−4.0 (3)
O2—C4—C5—C13	147.53 (18)	C13—C14—C15—C16	176.72 (18)
O2—C4—C5—C1	−32.3 (2)	C14—C15—C16—C17	0.2 (3)
C1—C2—C6—C7	178.79 (18)	C15—C16—C17—O4	−176.80 (18)
C3—C2—C6—C7	−1.9 (3)	C15—C16—C17—C18	4.0 (3)
C2—C6—C7—C8	162.9 (2)	O4—C17—C18—C19	176.59 (18)
C2—C6—C7—C12	−17.0 (3)	C16—C17—C18—C19	−4.2 (3)
C12—C7—C8—C9	2.8 (3)	C17—C18—C19—C14	0.2 (3)
C6—C7—C8—C9	−177.20 (17)	C15—C14—C19—C18	3.9 (3)
C7—C8—C9—C10	0.5 (3)	C13—C14—C19—C18	−176.94 (18)
C8—C9—C10—O3	176.46 (18)	C2—C3—O2—C4	−62.06 (19)
C8—C9—C10—C11	−3.4 (3)	C5—C4—O2—C3	62.96 (19)

### Hydrogen-bond geometry (Å, °)

**Table:**

*D*—H···*A*	*D*—H	H···*A*	*D*···*A*	*D*—H···*A*
O3—H3···O4^i^	0.82	1.95	2.757 (2)	167
O4—H4···O1^ii^	0.82	1.86	2.677 (2)	171

Symmetry codes: (i) −*x*+3/2, −*y*+2, *z*−1/2; (ii) −*x*+2, *y*+1/2, −*z*+3/2.
